# It's more than mechanics: the case for metabolic end-points in airways disease

**DOI:** 10.1183/13993003.01007-2026

**Published:** 2026-09-24

**Authors:** Chloe I. Bloom, Dinah Foer, Vanita R. Aroda, Katherine N. Cahill

**Affiliations:** 1 National Heart and Lung Institute, Imperial College London, London, UK; 2 Brigham and Women's Hospital, Harvard Medical School, Boston, MA, USA; 3 Vanderbilt University Medical Center, Nashville, TN, USA

## Abstract

**Metabolic dysfunction is a major, under-recognised driver of poor respiratory outcomes. Emerging evidence suggests it is a modifiable target that respiratory medicine can no longer afford to overlook.** https://bit.ly/4bmogqt

## Background

**FIGURE 1:**
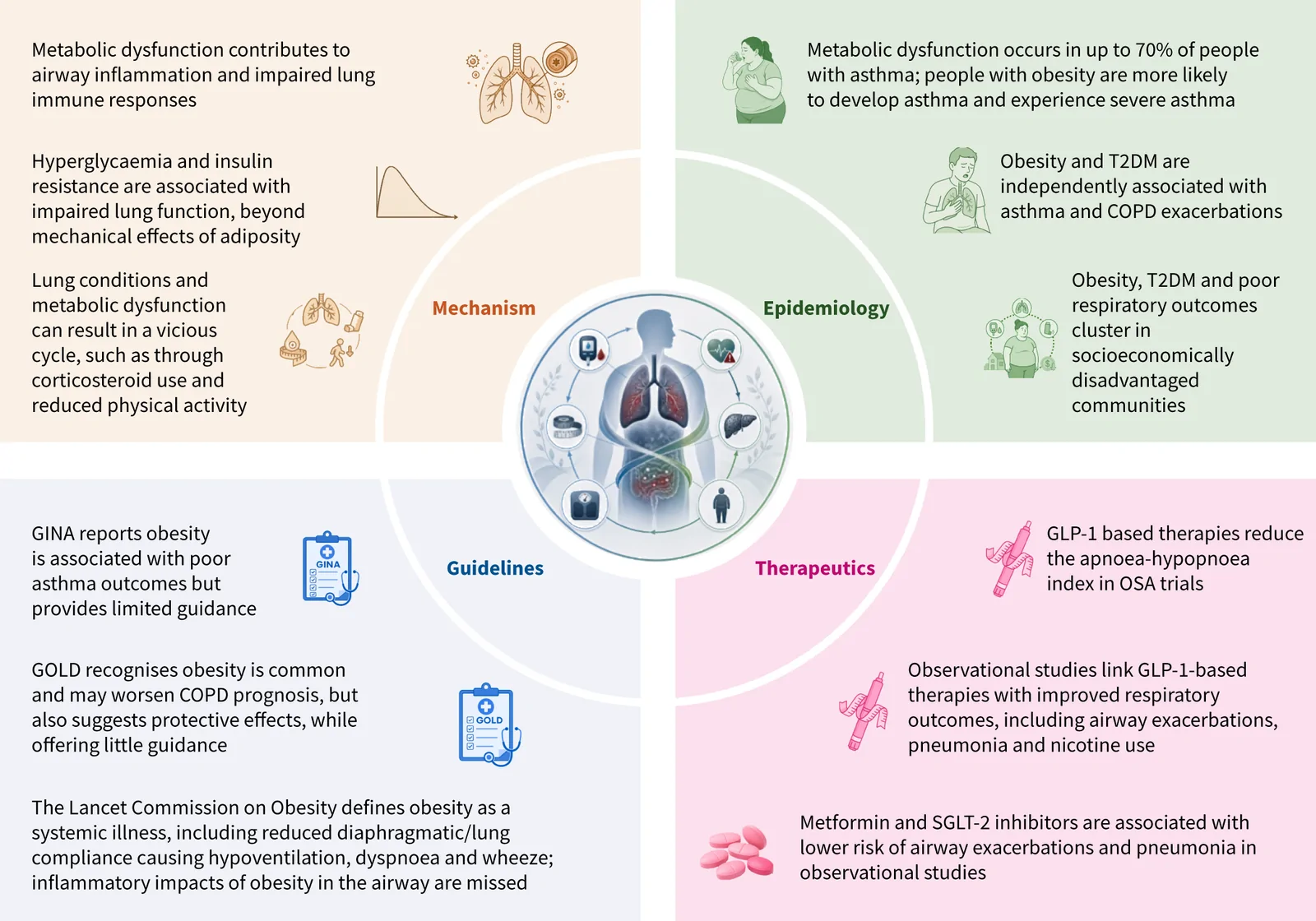
Evidence and evidence gaps linking metabolic dysfunction with respiratory health. T2DM: type 2 diabetes mellitus; GINA: Global Initiative for Asthma; GOLD: Global Initiative for Chronic Obstructive Lung Disease; GLP-1: glucagon-like peptide-1; OSA: obstructive sleep apnoea; SGLT-2: sodium-glucose cotransporter-2.

Metabolic dysfunction, including insulin resistance and hyperglycaemia, has been found to contribute independently to airway inflammation, impaired lung mechanics, and altered immune responses in the lungs (figure 1) [1–7]. Insulin resistance and compensatory hyperinsulinaemia can directly alter airway biology, including promoting airway smooth muscle proliferation and contractility, airway hyperresponsiveness, impaired beta-agonist responses, airway remodelling and inflammation [4, 7]. Hyperglycaemia may further contribute through oxidative stress, advanced glycation end-product/RAGE signalling, altered epithelial and immune responses, increased airway glucose and bacterial growth, and changes in lung connective tissue and compliance [4, 7]. Although metabolic dysfunction can occur at any body mass, these disturbances are more common in people with obesity and may act independently or synergistically with excess adiposity to worsen respiratory morbidity. Dyslipidaemia and altered adipokines, including FABP4, leptin and adiponectin, may also contribute to pulmonary vascular dysfunction, airway smooth muscle dysfunction, lung inflammation and airway hyperresponsiveness [7].

Up to 70% of people with asthma are overweight or obese, of whom around 10–20% have type 2 diabetes (T2DM), making obesity and associated metabolic dysfunction among the more common comorbidities in asthma [8, 9]. Mean body mass index (BMI) in adult asthma clinical trials and registries are consistently in the range of 28–30 kg·m^−2^ [8]. Higher BMI and glucose levels are associated with asthma exacerbations and poor asthma control, across type 2 low and type 2 high phenotypes [2, 4, 8]. Obesity may also reduce responsiveness to inhaled corticosteroids, further contributing to poor asthma control [5]. Encouragingly, an online weight-management intervention found that weight loss of only 5% or more was associated with clinically significant improvements in asthma control and quality of life, demonstrating that obesity is an important modifiable trait [10].

Similarly, in COPD, metabolic disease is associated with reduced lung function, more frequent exacerbations, and increased mortality [11, 12]. The prognostic importance of body mass in COPD is further demonstrated by its U-shaped association with mortality, with both underweight and obesity associated with increased all-cause, respiratory and cardiovascular mortality [13].

Diabetes is a well-recognised comorbidity in cystic fibrosis, where it is associated with poorer lung function and increased mortality. Emerging evidence also suggests that T2DM is an important high-risk feature in non-cystic fibrosis bronchiectasis, and is associated with more severe disease, increased infection risk, higher systemic inflammation and increased mortality [14].

This relationship can create a self-perpetuating cycle, whereby worsening airways disease leads to increased corticosteroid burden and reduced physical activity, further exacerbating metabolic dysfunction and obesity, and contributing to poor respiratory outcomes [15–17]. Respiratory patients are systematically under-assessed and under-treated for obesity and metabolic dysfunction, despite strong mechanistic links, high prevalence, and emerging evidence that metabolic therapies may significantly improve respiratory outcomes.

## The current narrow definition of obesity-related respiratory disease

Obstructive sleep apnoea (OSA) is often the only respiratory condition recognised as obesity-related, despite extensive epidemiological evidence, including observational and genetic studies, linking obesity to the development of asthma across the life course [3, 5, 18–20]. The relationship extends beyond the mechanical effects of obesity, suggesting a biological link between metabolic dysregulation and asthma pathophysiology.

The 2025 Lancet Commission on Obesity reached unanimous agreement that the diagnostic criteria for clinical obesity, a chronic systemic illness, should include respiratory manifestations [3]. Yet, these were limited solely to the mechanical effects of excess adiposity including hypoventilation, breathlessness and wheeze, attributed to reduced lung and diaphragmatic compliance, as well as apnoeas/hypopnoeas during sleep, due to increased upper airway resistance. Asthma was notably excluded from the criteria, despite the report acknowledging that asthma and obesity frequently co-occur.

Such reports reinforce a paradox: obesity is recognised as a major driver of respiratory morbidity, yet respiratory outcomes are rarely considered end-points in metabolic trials. Indeed, aside from OSA, respiratory conditions were not systematically captured in participants enrolled in the recent wave of glucagon-like peptide-1 receptor agonist (GLP-1 RA) trials, limiting the ability to conduct any respiratory-specific *post hoc* analyses. This may, in part, reflect the persistent underfunding of respiratory research [21].

## Emerging real-world evidence suggests benefit for respiratory patients

Obesity has become a global health crisis, affecting over one billion people and doubling in prevalence since 1990 [9]. If current trends continue, by 2050 over half of adults will be overweight or obese, and approximately 850 million to 1.3 billion people will be living with T2DM [22]. Fortunately, this unprecedented rise has been accompanied by a therapeutic revolution, with highly effective incretin-based therapies, originally developed to treat T2DM, now redefining the management of both obesity and T2DM. Clinical trials of these therapies, including GLP-1 RAs (*e.g.* dulaglutide, liraglutide, semaglutide) and the dual glucose-dependent insulinotropic polypeptide (GIP) and GLP-1 receptor co-agonist tirzepatide, have demonstrated benefits across multiple obesity-related conditions, including cardiovascular, liver and kidney disease, as well as osteoarthritis [23].

GLP-1 receptors are expressed in lung-resident cells, and GLP-1 RA signalling has been linked to anti-inflammatory and bronchodilatory pathways [24]. Preclinical and translational studies suggest GLP-1 receptor activation may reduce airway inflammation, including epithelial alarmins, NLRP3/IL-1β signalling and type 2 and non-type 2 responses, while promoting bronchodilation through cAMP-PKA signalling. Alongside this mechanistic rationale, there is also growing real-world evidence suggesting these therapies significantly reduce exacerbations of asthma and COPD and improve other respiratory outcomes, including pneumonia and nicotine addiction [25–32]. While the underlying mechanisms may differ across conditions, the consistency of these signals is sufficient to justify dedicated respiratory trials, and stratification of analyses by respiratory disease phenotypes and endotypes.

To date, however, the only completed respiratory trials of incretin-based therapies have focused on OSA. In particular, the phase 3 SURMOUNT-OSA programme showed clinically meaningful benefit with tirzepatide in adults with obesity and moderate-to-severe OSA [33], and these data supported subsequent US Food and Drug Administration approval for this indication. Additional respiratory trials are now underway in OSA, including retatrutide in the TRIUMPH programme (NCT05929066) and orforglipron in a dedicated master protocol (NCT06649045).

The lack of asthma-specific trials is difficult to justify, given the global burden of asthma and its strong association with obesity, and evidence indicating that bariatric surgery may improve asthma outcomes [34]. Initial asthma-focused studies have begun to emerge. These include GATA-3 (NCT05254314), an NIH-funded placebo-controlled trial evaluating whether semaglutide improves asthma control and reduces airway inflammation in obesity-associated asthma, and an Eli Lilly-sponsored international phase 2 trial (NCT07219173) evaluating whether brenipatide, a GIP/GLP-1 co-agonist, reduces exacerbations in moderate-to-severe asthma.

Alongside incretin-based therapies, there is also mounting evidence that other antidiabetic drugs, including metformin and sodium-glucose cotransporter-2 (SGLT-2) inhibitors, are associated with substantially fewer asthma and COPD exacerbations [27, 28, 35–37]. Metformin, a relatively inexpensive oral medication with a favourable safety profile, has received renewed attention in recent years with completed, ongoing and proposed large trials spanning ageing (TAME, MILES), cancer (STAMPEDE, MA.32), neurodegenerative disease (MAP, NCT07229651) and vascular disease (PERMET). Despite this, beyond a small two-centre US feasibility asthma trial (NCT06273072), a small asthma trial in Iran [38] and an earlier small COPD UK trial [39], there has been limited effort to evaluate metformin's effects on respiratory end-points.

## A missed opportunity: metabolic dysfunction in respiratory patients

In routine care, metabolic dysfunction is not systematically assessed in children with asthma or adults with asthma or COPD. For example, a UK primary care study found that around 40% of children with asthma had no recorded weight [40]. Given that obesity is a treatable trait in airway disease [41, 42], this represents a missed opportunity to improve respiratory outcomes and broader cardiometabolic outcomes.

The international asthma guidelines produced in 2026 by the Global Initiative for Asthma acknowledge obesity as a comorbidity associated with poor outcomes, yet largely non-specific recommendations are provided regarding its assessment and management [43]. This is due to a lack of evidence on how targeted treatments of obesity or metabolic dysfunction improve asthma outcomes. As a result, guidance focuses on general lifestyle advice rather than structured, evidence-based interventions, and metabolic assessment is not embedded within routine asthma management care [44].

The international COPD guidelines in the Global Initiative for Chronic Obstructive Lung Disease 2026 report likewise acknowledge obesity as a common comorbidity that may adversely affect prognosis [45]. However, the report also suggests that obesity may be protective against COPD progression and respiratory-related mortality, despite limited evidence to support or explain these apparently conflicting positions. Management recommendations remain limited to routine BMI monitoring and general lifestyle advice. Although “weight control medications” are mentioned, this is in the context of their systemic, rather than respiratory, effects. Together, these evidence gaps in the management of comorbid obesity in people with airways disease have likely contributed to therapeutic inertia and variation in clinical practice.

Moreover, current practice typically relies on BMI alone, which does not capture the metabolic heterogeneity that may shape disease risk and treatment response. For instance, insulin resistance has been linked to decline in lung function independently of the mechanical effects of excess body mass [2]. Respiratory research and clinical trials should move beyond BMI and incorporate metabolic markers to better characterise patients and identify those most likely to benefit from targeted interventions. Indeed, the 2025 Lancet Commission on Obesity suggests that excess adiposity should be confirmed by direct measurement of body fat or at least one anthropometric criterion (*e.g*. waist circumference, waist-to-hip ratio, or waist-to-height ratio), alongside BMI [3].

## A proposed path towards respiratory health in the metabolic era

**FIGURE 2:**
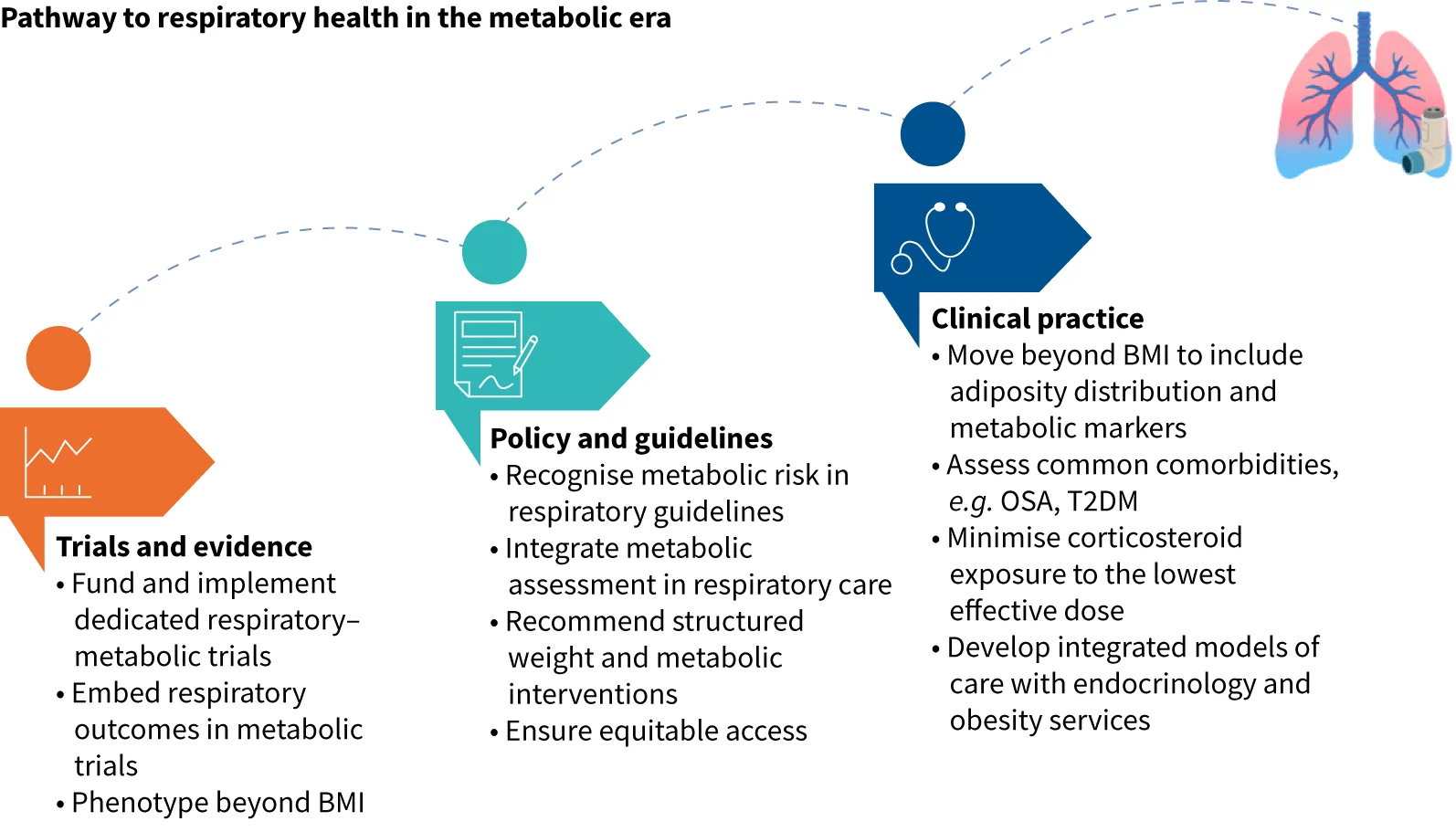
A proposed pathway towards better metabolic-respiratory health. BMI: body mass index; OSA: obstructive sleep apnoea; T2DM: type 2 diabetes mellitus.

In our view, future progress depends on evolution across three interconnected areas: trials and evidence generation, policy and guidelines, and clinical practice (figure 2).

### Trials and evidence generation

Caution is required in interpreting the current evidence base. Much of the apparent respiratory benefit of metabolic therapies comes from observational studies, and some reported effects could reflect weight loss, behavioural change, or residual confounding rather than direct effects on the airways. Moreover, not all patients with obesity have the same metabolic risk profile, and not all respiratory benefit is likely to be mediated through the same pathways. However, these uncertainties strengthen rather than weaken the case for dedicated respiratory trials: without them, the mechanisms, target populations and magnitude of benefit will remain unclear.

Importantly, respiratory outcomes should be studied not only in established respiratory disease, but also in people with obesity and type 2 diabetes without diagnosed lung disease. This would establish the lung as a site of end-organ dysfunction in metabolic disease and clarify whether metabolic therapies can preserve lung health before overt respiratory disease develops.

Respiratory outcomes should therefore be incorporated routinely into metabolic therapy trials, just as cardiovascular, renal and hepatic outcomes are now commonly included. Relevant respiratory end-points could include asthma and COPD exacerbations, changes in lung function, changes in type 2 inflammation biomarkers (*e.g.* exhaled nitric oxide fraction, blood eosinophil counts), smoking cessation, respiratory infection, and patient-reported respiratory outcomes.

Conversely, respiratory trials should include more detailed metabolic phenotyping, extending beyond BMI alone [46]. In addition, subgroup analyses should be prespecified to assess heterogeneity of treatment effect according to metabolic phenotype, as well as respiratory disease phenotype and endotype.

### Policy and guidelines

It is understandable that guideline committees may be reluctant to make strong recommendations in the absence of prospective trial data. However, leaving respiratory–metabolic risk insufficiently recognised in the interim also carries consequences. Without explicit acknowledgement of these links, opportunities for earlier identification, risk stratification, and integrated care may be missed, and the field remains stuck in a cycle in which limited recognition contributes to limited evidence generation.

Common respiratory diseases should therefore be explicitly recognised as conditions closely linked to obesity and metabolic dysfunction, rather than being viewed through the narrow lens of mechanical complications. Respiratory guidelines should better reflect the importance of metabolic assessment and intervention in airway disease, while metabolic guidelines should acknowledge the lungs as a site of end-organ dysfunction. This does not require guidelines to mandate specific interventions before definitive trial evidence is available, but it does require recognition that these links are clinically relevant and worthy of attention in research and practice. Such acknowledgement would also strengthen the case for funding prospective studies needed to support firmer recommendations.

Equitable access to effective weight and metabolic interventions must also be addressed. Metabolic dysfunction and adverse respiratory outcomes frequently occur in socioeconomically disadvantaged communities, yet access to dietetic support, structured weight management services, pulmonary rehabilitation, and newer metabolic therapies is often most limited in those communities [47, 48]. Any future respiratory–metabolic strategy must therefore address not only what treatments are effective, but who is able to receive them.

In our view, greater recognition at policy and guideline level is essential not only to improve care, but also to recognise the emerging importance of respiratory–metabolic research and its associated evidence base.

### Clinical practice

Metabolic assessment could be embedded within routine respiratory care pathways in both primary and specialist care for adults and, where appropriate, for children. At a minimum, basic measures such as weight should be recorded consistently, reinforcing that metabolic health is an important component of the assessment and management of respiratory disease. Although not a traditional vital sign, BMI has long been proposed as one, reflecting its value as an indicator of health and predictor of future morbidity and mortality at the population level [49, 50].

In specialist settings, assessment could extend beyond BMI alone to include measures of adiposity distribution, such as waist circumference or waist-to-height ratio [51], together with metabolic markers including glucose, HbA1c, lipid profile and insulin resistance. There could be systematic evaluation for common comorbidities, including OSA, T2DM, metabolic dysfunction-associated steatotic liver disease, and hypertension. Corticosteroid exposure remains a source of metabolic risk for patients with asthma, and steroid-sparing options should be prioritised [52]. While most of the respiratory biologics are steroid-sparing, patients with obesity may be less likely to receive these medications, despite evidence that elevated BMI does not affect treatment response [53–55]. Patients and healthcare professionals alike should be aware that corticosteroids' well-established benefits on the airways may come at a metabolic cost.

More integrated models of care are needed, with shared pathways linking respiratory medicine, primary care, endocrinology, and obesity services. Mass General Brigham in Boston, MA, USA has already established a metabolic asthma clinic, providing an early example of an integrated service model that could become more widely available.

## Conclusion

Respiratory societies, guideline committees, trialists, funders, regulators and industry all have a role in recognising the close relationship between respiratory and metabolic disease, and incorporating respiratory outcomes within the metabolic agenda. Unless respiratory disease is deliberately embedded into metabolic research, service design and policy – and, equally, metabolic parameters are embedded within respiratory research – the field risks missing potentially the most important therapeutic shift affecting airway disease since biologics.
